# The best QT correction formula in a non-hospitalized population: the Fasa PERSIAN cohort study

**DOI:** 10.1186/s12872-022-02502-2

**Published:** 2022-02-16

**Authors:** Mohammad Hosein Yazdanpanah, Mohammad Mehdi Naghizadeh, Sepideh Sayyadipoor, Mojtaba Farjam

**Affiliations:** 1 Noncommunicable Diseases Research Center, Fasa University of Medical Sciences, Ibn-Sina Square, P.O. Box: 74616-86688, Fasa, Fars Iran; 2 Liver and Pancreatobiliary Diseases Research Center, Digestive Diseases Research Institute, Tehran University of Medical Sciences, Tehran, Iran; 3 Student Research Committee, Fasa University of Medical Sciences, Fasa, Iran

**Keywords:** QT interval, QTc prolongation, QT correction formula, Survival, Iran

## Abstract

**Background:**

QT interval as an indicator of ventricular repolarization is a clinically important parameter on an electrocardiogram (ECG). QT prolongation predisposes individuals to different ventricular arrhythmias and sudden cardiac death. The current study aimed to identify the best heart rate corrected QT interval for a non-hospitalized Iranian population based on cardiovascular mortality.

**Methods:**

Using Fasa PERSIAN cohort study data, this study enrolled 7071 subjects aged 35–70 years. Corrected QT intervals (QTc) were calculated by the QT interval measured by Cardiax® software from ECGs and 6 different correction formulas (Bazett, Fridericia, Dmitrienko, Framingham, Hodges, and Rautaharju). Mortality status was checked using an annual telephone-based follow-up and a minimum 3-year follow-up for each participant. Bland–Altman, QTc/RR regression, sensitivity analysis, and Cox regression were performed in IBM SPSS Statistics v23 to find the best QT. Also, for calculating the upper and lower limits of normal of different QT correction formulas, 3952 healthy subjects were selected.

**Results:**

In this study, 56.4% of participants were female, and the mean age was 48.60 ± 9.35 years. Age, heart rate in females, and QT interval in males were significantly higher. The smallest slopes of QTc/RR analysis were related to Fridericia in males and Rautaharju followed by Fridericia in females. Thus, Fridericia’s formula was identified as the best mathematical formula and Bazett’s as the worst in males. In the sensitivity analysis, however, Bazett’s formula had the highest sensitivity (23.07%) among all others in cardiac mortality. Also, in the Cox regression analysis, Bazett’s formula was better than Fridericia’s and was identified as the best significant cardiac mortality predictor (Hazard ratio: 4.31, 95% CI 1.73–10.74, *p* value = 0.002).

**Conclusion:**

Fridericia was the best correction formula based on mathematical methods. Bazett’s formula despite its poorest performance in mathematical methods, was the best one for cardiac mortality prediction. Practically, it is suggested that physicians use QTcB for a better evaluation of cardiac mortality risk. However, in population-based studies, QTcFri might be the one to be used by researchers.

**Supplementary Information:**

The online version contains supplementary material available at 10.1186/s12872-022-02502-2.

## Background

The QT interval on an electrocardiogram (ECG) indicates ventricular repolarization which is measured from the beginning of the QRS wave to the end of the T wave [1]. It is an important parameter on ECG due to its prolongation.

Prolongation of QT has two main categories of etiology. The first is a congenital or familial form of QT prolongation, known as long QT syndrome (LQTS). There are at least 14 genes responsible for LQTS [2], and the prevalence of LQTS is reported as approximately one in 2500 people [3] which makes this etiology an important one. The second is an acquired form of QT prolongation. The role of several factors such as smoking [4], elevated lipid profiles [5], a history of prior cardiovascular diseases [6], chronic diseases such as diabetes, renal failure, and thyroid disturbances [7], metabolic syndrome [8], body fat mass [9], liver failure [10], electrolyte imbalances like potassium [11] and calcium [7], as well as that of some medications, including antimicrobial, psychoactive, and cardiovascular drugs, have been shown and suggested in QT prolongation [12]. Besides, the effects of age [13] and the female gender [14] on the QT interval should not be ignored.

QT prolongation can cause clinical symptoms such as vertigo and syncope [15] and may lead to cardiovascular events such as stroke and ventricular arrhythmias, including ventricular fibrillation (VF), Torsade de Pointes (TdP) [16, 17], and ultimately, sudden cardiac death (SCD) [18]. Recently, Simpson et al. showed the association between drug-induced QT prolongation and SCD [19], showing an increased risk of non-arrhythmic causes of death. Prolongation of the QT interval has been studied extensively in patients with cardiac diseases, and its association with increased risk of all-cause mortality has been reported [20].

The relation between QT and RR intervals has been reported to be highly individual [21, 22]. Also, it has shown that heterogeneity of myocardial repolarization will not change with increasing age [23]. It is not clear why the QT/RR relationship should reflect interindividual variability physiologically, but it is contingent on the complex interplay of the individual ionic channels which hold the potential for ventricular myocyte action [24, 25]. Moreover, the genes responsible for these channels may vary among individuals [26, 27]. In this case, the ionic complexity of the repolarization process could perhaps lead to major differences, even between healthy normal hearts [21, 28]. There are various formulas for the calculation of the corrected QT interval to adjust the heart rate effect and cause the relationship between RR and QT interval to subside [29]. Formulas known for QT interval correction are presented in Table 1. The most popular correction formula was proposed by Bazett (QTcB) [30]. According to the study by Shen Luo et al. [31], Bazett’s formula has only become popular because physicians recommend it to their students who lack knowledge of the benefits of other formulas and use it merely as instructed. They have also suggested that Hodges’ formula is the best correction formula in the field. Lately, Dash et al. [32] reported that Fridericia’s formula may be a better choice than Bazett’s for the calculation of QTc in subjects with AF.

**Table 1:** Different QT correction formulas

Corrections	Formulas
Exponential	
Bazett [30] (QTcB)	QTcB = QT/RR0.50
Fridericia [79] (QTcFri)	QTcFri = QT/RR0.333
Dmitrienko [80] (QTcD)	QTcD = QT/RR0.413
Linear	
Framingham [37] (QTcFra)	QTcFra = QT + 0.154 (1-RR)
Hodges [38] (QTcH)	QTcH = QT + 0.00175 (HR-60)
Rautaharju [81] (QTcR)	QTcR = QT − 0.185 (RR-1) + k^a^

*QT* QT interval (ms), *RR* RR interval (ms), *HR* heart rate (bpm)

^a^k =  + 6 ms for men and + 0 ms for women

The current study aimed to identify the best heart rate corrected QT interval for a non-hospitalized Iranian population to determine which QT correction formula would be the best based on cardiovascular-cause mortality among Fasa PERSIAN cohort study subjects.

## Method

### Population

Data from the Fasa Cohort Study as a branch of the PERSIAN cohort which was started on 2015–2016 was used [33]. All participants came from a rural region in Fasa and within the age range of 35–70 years old. Before entering the Fasa Cohort Study, each participant signed an informed consent letter. A telephone-based follow-up was performed each year for all of the participants without any missing data. In the case of an event, their relatives were interviewed about it. Patients' medical data were checked and analyzed in cases of hospitalization or death. Each participant with a recorded electrocardiogram (ECG) entered the study (n = 7239). Subjects with incomplete ECG data and those with a non-sinus rhythm ECG or QRS duration of more than 120 ms were excluded (n = 168). Ultimately, 7071 subjects remained in the study.

### Demographics and medical history

Demographic data regarding age and gender were recorded in a questionnaire. Patients’ histories of cardiovascular diseases (CVD) such as stroke, coronary heart disease (CHD), or myocardial infarction (MI) as well as medical conditions such as diabetes, hypertension, renal failure, chronic lung disease, hepatitis, and thyroid dysfunction were questioned and evaluated by the protocol mentioned in reference 32 [33]. Patients were asked to bring any medication taken within 2 weeks of the study for registration at the interview time for maximum precision and to be recorded in electrical form. Mortality status was checked using a telephone-based follow-up each year, and the date of a subject’s mortality was recorded electronically. National and regional death registry systems were searched for confirmation and the reason for death.

### Electrocardiogram

Each participant had a 12-lead ECG with a 2000 Hz sampling rate and 0.04 µV/bit (24-bit resolution). ECGs were recorded using a device (Cardiax® [34]) on shaved precordium and with patients in the supine and postprandial state for the best possible results. Patients were in the supine position 15 min before recording and were told to relax, breathe normally, refrain from moving and talking, but remain awake during the procedure. Heart rates, QRS duration, and QT intervals of all ECGs were analyzed and reported automatically by the Cardiax software (version 3.50.2, International Medical Equipment Developing Co. Ltd., Budapest, Hungary) and exported to the central data software. The mean of QT intervals on lead II on every beat during 10 s was reported as the QT interval of each subject. For the correction of QT intervals, the 6 most popular correction formulas were used (Table 1).

### Normal healthy subjects for optimum QT correction formula

To find the best QT correction formula, first, the healthy subjects in the study were selected for the best results. Individuals with the following criteria were selected: (1) having no CVD history; (2) having no significant medical condition, such as diabetes, hypertension, renal failure, chronic lung disease, hepatitis, or thyroid dysfunction; (3) no medication consumption that would influence the cardiovascular system and the QT interval; (4) having an RR interval of 0.5–1.5 s.

The American organization CredibleMeds provides lists of drugs that are associated with QTc-prolongation which are continuously updated with new information and revised by their review team. This study used a list of drugs with known and possible risks for TdP, which accounted for 187 cardiac and non-cardiac drugs in total [35].

Finally, 3952 subjects were identified as meeting the above criteria, and their data were used in analyses to find the optimum QT correction formula. Their data was also used in calculating the upper and lower limits of normal (ULN and LLN) for each QT correction formula based on the 95% and 5% percentile of these subjects, respectively.

### Statistical analysis

Data were presented as mean ± standard deviation (SD) and count (percentage) and were compared between males and females by independent sample *t* and chi-square tests. The paired *t* test was used to compare mean differences of QTc intervals, and their intercorrelation was evaluated by the Pearson correlation coefficient. To indicate the differences between QTc intervals, a Bland–Altman plot was used [36]. Four different methods were used to find the optimum QT correction formula. The QTc_1_ was a linear regression model between QT and RR adjusted by gender based on the Framingham formula (QT = α + β * RR – β_2 _* male) [37]. The QTc_2_ was another linear regression model between QT and heart rate based on the Hodges formula (QT = α + β * HR) [38]. The QTc_3_ was a logarithmic transformation of both QT and RR based on Spence et al. [39] after gender distinction. The slope (β) of the linear regression between QT interval and RR after logarithmic transformation was obtained (LogQT = α + β * LogRR), and then the optimized formula was defined as (QTc_3_ = QT/RR^β^). The fourth method was based on Wernicke et al. [40]. The value of “*d*” was found to range from 0.301 to 0.499 by 0.001 in QTc_4_ = QT/RR^d^ as the lowest correlation between QTc_4_ and RR. A simple linear regression separated by gender between the RR and QTc intervals was performed to find the best correction formula for the study population. The calculations for ULN and LLN and their 95% confidence interval have been described above. To validate the ULN cut points which were extracted from normal healthy population QTc intervals, sensitivity, specificity, positive predictive value (PPV), and negative predictive value (NPV) were calculated for the prediction of all-cause and cardiac mortality rates. Cox regression analysis was performed to calculate the hazard ratio (HR) and 95% confidence interval of mortality. In this analysis, survival days to death as time, death as an event, and QTc interval > ULN as an independent factor were defined. Cox regression analysis was performed in both unadjusted and multivariable-adjusted models.

## Results

### Demographics

From 7071 subjects, 56.4% were female, and the mean age was 48.60 ± 9.35 years. Age and heart rate in females and QRS duration and QT interval in males were significantly higher (*p* < 0.001). After the correction of the QT interval, all QTc interval means were higher in females (*p* < 0.001). Cardiac deaths and all-cause mortality were more frequent in male subjects. The baseline characteristics of subjects are reported in Table 2 according to gender. Other descriptive data on ECG parameters are reported in Additional file 1: Table S1.

**Table 2:** Baseline characteristics of subjects according to gender

Variables	Male n = 3078	Female n = 3993	*p* value
Age (years)	48.49 ± 9.28	48.69 ± 9.40	0.367^a^
Heart rate (bpm)	65.99 ± 10.87	75.16 ± 11.59	**< 0.001**^**a**^
QRS duration (ms)	98.50 ± 10.70	95.46 ± 9.90	**< 0.001**^**a**^
QT interval (ms)	403.82 ± 36.32	395.63 ± 37.19	**< 0.001**^**a**^
QTcB (ms)	420.24 ± 31.91	439.63 ± 33.58	**< 0.001**^**a**^
QTcFri (ms)	414.40 ± 29.57	424.05 ± 32.63	**< 0.001**^**a**^
QTcFra (ms)	414.09 ± 29.14	423.83 ± 30.36	**< 0.001**^**a**^
QTcH (ms)	414.30 ± 29.33	422.16 ± 29.92	**< 0.001**^**a**^
QTcR (ms)	422.16 ± 29.75	429.40 ± 31.05	**< 0.001**^**a**^
QTcD (ms)	417.14 ± 30.18	431.46 ± 32.15	**< 0.001**^**a**^
All-cause mortality	46 (1.5)	33 (0.8)	**0.008**^**b**^
Cardiac mortality	26 (0.8)	19 (0.5)	0.053^**b**^
Survival days^+^	1154.27 ± 115.23	1172.65 ± 107.32	**< 0.001**^**a**^
Subjects with mortality	600.08 ± 312.08	655.51 ± 346.86	**< 0.001**^**a**^
Subjects without mortality	1162.68 ± 85.45	1176.96 ± 91.61	**< 0.001**^**a**^
Selected medications†	691 (22.4)	1124 (28.1)	**< 0.001**^**b**^
CVD history	302 (9.8)	557 (13.9)	**< 0.001**^**b**^
Stroke	40 (1.3)	52 (1.3)	0.992^**b**^
CHD	274 (8.9)	522 (13.1)	**< 0.001**^**b**^
MI	77 (2.5)	52 (1.3)	**< 0.001**^**b**^
Medical conditions	617 (20.0)	1773 (44.4)	**< 0.001**^**b**^
Diabetes	251 (8.2)	638 (16.0)	**< 0.001**^**b**^
Hypertension	354 (11.5)	1083 (27.1)	** < 0.001 **^**b**^
Renal failure	35 (1.1)	33 (0.8)	0.184^**b**^
Chronic lung disease	50 (1.6)	85 (2.1)	0.124^**b**^
Hepatitis	3 (0.1)	0 (0.0)	**0.048**^**b**^
Thyroid dysfunction	76 (2.5)	563 (14.1)	**< 0.001**^**b**^

Data are reported as mean ± standard deviation or as count (percentages). *p* value reported as the result of ^a^Independent sample t and ^b^Chi-square tests. Statistically significant *p* values are bolded (*p* value < 0.05)

*bpm* beats per minute, *QTc* corrected QT interval, Bazett’s correction formula (QTcB), Fridericia’s correction formula (QTcFri), Dmitrienko’s correction formula (QTcD), Framingham’s correction formula (QTcFra), Hodges’s correction formula (QTcH), Rautaharju’s correction formula (QTcR), *ms* milliseconds

^+^Survival days calculated from the day of electrocardiogram recording until their last follow-up in Fasa PERSIAN cohort Study

^†^Selected medications is a list of drugs with known and possible risks for Torsade de Pointes, which accounted for 187 cardiac and non-cardiac drugs in total [35]

### Mean difference and correlation of different QTc intervals

The highest correlation was detected between Framingham and Fridericia QTc intervals (r = 0.995, *p* value < 0.001), and the smallest correlation was related to Bazett-Fridericia and Hodges-Rautahatju QTc intervals (r = 0.935, *p* value < 0.001) in male subjects. In females, interestingly the highest correlation was r = 0.992 in Rautahatju and Dmitrienko QTc intervals with *p* value < 0.001, and the smallest correlation, which was the same as in male subjects, was found in the Bazett and Fridericia QTc intervals (r = 0.918, *p* value < 0.001). All mean differences and correlations between different QTc formulas in both genders have been reported in Additional file 1: Table S2. For a better comparison of different QTc intervals, the Bland–Altman analysis was performed (Additional file 1: Table S3). The Bland–Altman graphs in males and the Bland–Altman graphs for females is provided in Additional file 1: Figure S1.

### The optimum QT correction formula

To determine the best QT correction formula for our study population, four statistical methods from previously published papers were evaluated. The similar statistical method from 4 common QT correction formulas performed and their results are reported in Table 3. First, a method similar to the Framingham formula was used, a linear regression model between QT interval and RR. The results showed QT = 0.267 + 0.155 * RR − 0.009 * male, which was very similar to the Framingham correction formula (QT = 0.234 + 0.154 * RR − 0.012 * male) [37]. For the second method, heart rate was used in a linear regression model with a QT interval similar to that of the Hodges formula. The data showed the slope of this regression to be 0.176 (QT = 0.176 * HR), which was very near the Hodges correction formula (QTcH = QT + 0.00175 (HR-60)) [38]. The third method used was one previously described in Spence et al. [39]. β for females and males in this method was 0.365 (standard error = 0.011) and 0.333 (standard error = 0.011), respectively. The male slope had the same coefficient in the Fridericia formula, and the female slope was only slightly higher. The final method used was introduced by Wernicke et al. [40]. The value of “d” was considered to be the result of the zero correlation between the QT and RR intervals (d = 0.316). Again, the correction formula was similar to the Fridericia formula. The R^2^ of these correction formulas is reported in Table 3.

**Table 3:** Determination of the best qt correction formula based on previously published equations

QT correction model	Description	QT correction formula		R^2^
Similar to Framingham Heart study [37]	Linear regression model	QTc_1_ = QT + 0.155 * RR − 0.009 * Gender		0.347
Similar to Hodges study [38]	Linear regression model	QTc_2_ = QT + 0.176 * RR		0.343
Similar to Spence et al. [39]	Linear regression model after logarithmic transformation	Female	QTc_3_ = QT/RR**0.365	0.346
		Male	QTc_3_ = QT/RR**0.333	0.376
Similar to Wernicke et al. [40]	Finding zero correlation between QTc and RR	QTc_4_ = QT/RR**0.316		–

*QT* QT interval (s), *RR* RR interval (s)

Ultimately, attempts to find the best QT correction formula for the current study population led to three previously published formulas, the Framingham, Hodges, and Fridericia formulas. As reported in Additional file 1: Tables S2 and Table 3, these three formulas are closely correlated; thus, a QTc and RR regression analysis was performed to achieve a better comparison.

### QTc and RR regression analysis

The best QTc formula is the one that minimizes the influence of RR in the QTc interval. Therefore, when a scatter-dot plot between QTc and RR is drawn, the formula with the lowest slope and R^2^ closest to zero is the best correction formula.

**Fig. 1:**
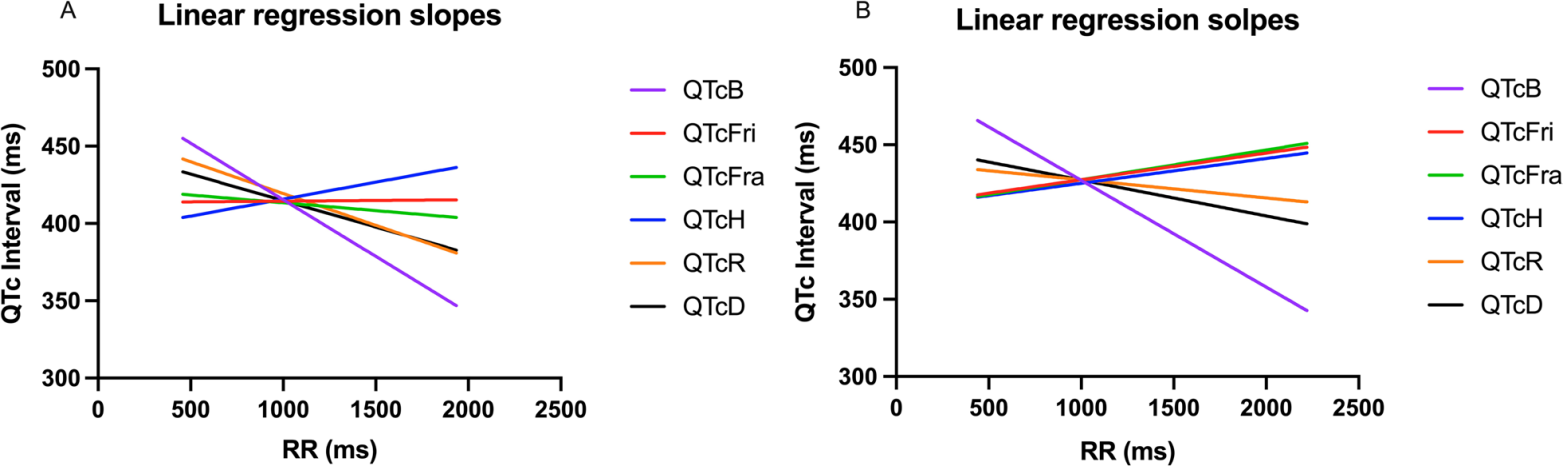
Comparison of linear regression slopes of different QTc formulas in both genders. **A** QTc/RR analysis in male, **B** QTc/RR analysis in females. *QTc* corrected QT interval, *RR* RR interval, Bazett’s correction formula (QTcB), Fridericia’s correction formula (QTcFri), Dmitrienko’s correction formula (QTcD), Framingham’s correction formula (QTcFra), Hodges’s correction formula (QTcH), Rautaharju’s correction formula (QTcR)

The data from the QTc/RR analysis of each QT correction formula is shown in Table 4. In males, the smallest slope and R^2^ were related to QTcFri. In females, QTcR had the smallest slope and R^2^ followed by QTcFri and QTcH. For a better visual comparison, Fig. 1 has been provided. In this figure, a straighter line means the minimum influence of RR in QTc.

**Table 4:** Determination of the lowest beta coefficient to find the best correction formula among previously published formulas by linear regression analysis of QTc and RR according to both genders

QTc formulas	B for RR	95% CI	Constant (ms)	95% CI (ms)	R^2^
Male					
QTcB	− 0.073	− 0.080 to − 0.066	488.42	481.79 to 495.058	0.119
QTcFri	0.001	− 0.006 to 0.008	413.41	406.86 to 419.96	< 0.001
QTcFra	− 0.10	− 0.017 to 0.003	423.42	416.98 to 429.87	0.003
QTcH	0.022	0.015 to 0.029	393.68	387.22 to 400.13	0.013
QTcR	− 0.041	− 0.048 to 0.034	460.42	453.98 to 466.87	0.043
QTcD	− 0.034	− 0.041 to − 0.027	448.99	442.41 to 455.58	0.029
Female					
QTcB	− 0.069	− 0.077 to − 0.061	495.97	489.30 to 502.64	0.066
QTcFri	0.016	0.008 to 0.024	410.71	404.01 to 417.40	0.004
QTcFra	0.019	0.012 to 0.027	408.09	401.86 to 414.31	0.006
QTcH	0.016	0.009 to 0.023	409.04	402.90 to 415.18	0.004
QTcR	− 0.012	− 0.020 to 0.004	439.20	432.82 to 445.58	0.002
QTcD	− 0.023	− 0.031 to − 0.015	450.40	443.82 to 456.99	0.008

*QTc* corrected QT interval, *RR* RR interval, *95% CI* 95% confidence interval, *ms* milliseconds, Bazett’s correction formula (QTcB), Fridericia’s correction formula (QTcFri), Dmitrienko’s correction formula (QTcD), Framingham’s correction formula (QTcFra), Hodges’s correction formula (QTcH), Rautaharju’s correction formula (QTcR)

### The normal limits

The healthy subjects in the current study were n = 3952. The highest ULN (458.92 and 476.84 ms in males and females, respectively) among the QTc was related to Bazett in both genders. As expected, there were differences between males and females in ULN and LLN. Overall, LLNs and ULNs were higher in women. All LLNs and ULNs of each QTc interval are reported in Table 5.

**Table 5:** The normal limits of different QTc intervals in both genders

QTc interval	LLN (ms)	95% CI (ms)	ULN (ms)	95% CI (ms)
Male n = 2028				
QTcB	379.13	377.09–381.12	458.92	455.36–462.28
QTcFri	378.85	376.12–380.13	448.84	446.23–450.88
QTcFra	377.81	375.54–380.00	448.00	445.33–450.00
QTcH	379.25	377.50–381.00	449.38	446.50–452.75
QTcR	384.49	382.47–386.47	456.63	453.97–459.56
QTcD	379.06	377.10–381.34	452.38	449.19–456.13
Female n = 1924				
QTcB	398.21	396.19–400.28	476.84	473.43–480.36
QTcFri	386.09	383.45–389.04	457.64	454.20–461.65
QTcFra	387.80	386.09–390.67	456.08	452.52–458.67
QTcH	387.06	384.75–389.50	452.93	450.50–456.25
QTcR	394.69	391.64–396.49	460.66	456.84–465.00
QTcD	393.39	389.86–395.26	465.02	461.47–468.90

*QTc* corrected QT interval, *LLN* lower limit of normal, *ULN* upper limit of normal, *95% CI* 95% confidence interval, *ms* milliseconds, Bazett’s correction formula (QTcB), Fridericia’s correction formula (QTcFri), Dmitrienko’s correction formula (QTcD), Framingham’s correction formula (QTcFra), Hodges’s correction formula (QTcH), Rautaharju’s correction formula (QTcR)

### Validation of QTc intervals by mortality

Among the total study population, 79 (1.1%) subjects died within 3 years from registration day, of whom 58% were male and 56% of deaths were due to cardiac causes. Table 6 shows the sensitivity, specificity, PPV, and NPV for all-cause and cardiac mortality predictions for subjects with a QTc higher than ULN in males. In the case of all-cause mortality, the sensitivity levels of QTcB, QTcFra, QTcR, and QTcD were higher than the others (17.39%) with almost equal specificity (~ 93%). In the case of cardiac mortality, QTcB with a sensitivity of 23.07% was the most sensitive, followed by QTcFri, QTcFra, QTcR, and QTcD with a sensitivity level of 15.38% and almost equal specificity (~ 93%). Information about sensitivity, specificity, PPV, and NPV for all-cause and cardiac mortality predictions in females is reported in Additional file 1: Table S4 due to low index values.

**Table 6:** Sensitivity analysis of all-cause and cardiac mortality prediction by QTc > ULN in different QTc intervals in males

QTc intervals		All-cause mortality				Cardiac mortality			
	QTc > ULN (%)	Sens (%)	Spec (%)	NPV (%)	PPV (%)	Sens (%)	Spec (%)	NPV (%)	PPV (%)
QTcB	6.6	17.39	93.56	98.67	3.94	23.07	93.54	99.30	2.95
QTcFri	6.5	15.21	93.66	98.64	3.51	15.38	93.61	99.23	2.01
QTcFra	6.3	17.39	93.89	98.68	4.14	15.38	93.80	99.23	2.07
QTcH	6.2	10.86	93.83	98.57	2.60	11.53	93.80	99.20	1.56
QTcR	6.4	17.39	93.76	98.68	4.06	15.38	93.67	99.23	2.03
QTcD	6.5	17.39	93.66	98.67	4.00	15.38	93.57	99.23	2.00

*ULN* upper limit of normal, *QTc* corrected QT interval, *Sens* sensitivity, *Spec* specificity, *PPV* positive predictive value, and *NPV* negative predictive value, Bazett’s correction formula (QTcB), Fridericia’s correction formula (QTcFri), Dmitrienko’s correction formula (QTcD), Framingham’s correction formula (QTcFra), Hodges’s correction formula (QTcH), Rautaharju’s correction formula (QTcR)

### Cox regression analysis

A summary of the Cox regression analysis is reported in Table 7. In the unadjusted regression, the HRs of QTcB, QTcFri, QTcFra, QTcR, and QTcD were statistically significant. The highest HR for all-cause mortality was related to QTcFra (HR = 3.20, *p* value = 0.003). In cardiac mortality, the highest and only significant HR was related to QTcB (HR = 4.31, *p* value = 0.002). After performing adjusted model analysis with age and heart rate, the highest HR values decreased, yet they both remained statistically significant (*p* value < 0.05). The results of the Cox regression analysis in female subjects and both genders are reported in Additional file 1: Table S5 and S6. Also, the detailed result of the Cox regression analysis of the un- and multivariate-adjusted model of All-cause and cardiac mortality prediction by QTc > ULN in different QTc intervals in males are reported in Additional file 1: Table S7. One single Cox regression model was performed to allow all QTcs to compete with each other (Additional file 1: Table S8). In this regression analysis, age and heart rate (as continues variables) were forced to stay in the model in all steps. Six correction formulas were competing with each other as a backward stepwise selection variable method. There are two separate regression models for all-cause and CVD mortality. QTcFra in the all-cause mortality model and QTcB in the cardiac-cause mortality model was only remaining in the final step (*p* value < 0.05).

**Table 7:** Cox regression analysis of the un- and multivariate-adjusted model of all-cause and cardiac mortality prediction by QTc > ULN in different QTc intervals in males

	Unadjusted						Multivariate-adjusted					
	All-cause mortality			Cardiac mortality			All-cause mortality			Cardiac mortality		
	HR	95%CI	*p* value	HR	95%CI	*p* value	HR	95%CI	*p* value	HR	95%CI	*p* value
QTcB	**3.02**	**1.41–6.49**	**0.004**	**4.31**	**1.73–10.74**	**0.002**	**2.36**	**1.06–5.28**	**0.036**	**2.92**	**1.09–7.78**	**0.032**
QTcFri	**2.63**	**1.18–5.89**	**0.018**	2.67	0.92–7.76	0.070	**2.52**	**1.13**–**5.64**	**0.025**	2.46	0.85–7.19	0.096
QTcFra	**3.20**	**1.49**–**6.87**	**0.003**	2.77	0.95–8.05	0.060	**3.08**	**1.43**–**6.62**	**0.004**	2.59	0.89–7.54	0.081
QTcH	1.84	0.73–4.67	0.195	1.97	0.59–6.59	0.266	1.92	0.75–4.90	0.171	2.08	0.62–6.99	0.236
QTcR	**3.13**	**1.46**–**6.71**	**0.003**	2.70	0.93–7.85	0.067	**2.84**	**1.32**–**6.09**	**0.007**	2.32	0.80–6.40	0.122
QTcD	**3.08**	**1.43**–**6.60**	**0.004**	2.66	0.91–7.72	0.072	**2.65**	**1.23**–**5.72**	**0.013**	2.09	0.71–6.10	0.179

*ULN* upper limit of normal, *HR* Hazard ratio, *CI* confidence interval, statistically significant HRs are bolded. *QTc* Corrected QT interval, Bazett’s correction formula (QTcB), Fridericia’s correction formula (QTcFri), Dmitrienko’s correction formula (QTcD), Framingham’s correction formula (QTcFra), Hodges’s correction formula (QTcH), Rautaharju’s correction formula (QTcR). Multivariate-adjust was done with age and heart rate

## Discussion

### Main findings

The findings of the current study suggest that (1) the best mathematical rate correction QT interval formula is the QTcFri, and (2) QTcB may be the best QT correction formula based on cardiac mortality. (3) Also, our study suggested QTcFra as the best QT correction formula based on all-cause mortality. Attempts to find the best QT correction for the non-hospitalized population in this study led us to the QTcFra, QTcH, and QTcFri formulas. For a better comparison of these three formulas, different statistical analyses were performed, and QTcFri was identified as the best formula for the current study population. To validate it in clinical aspects, QTcFri was surprisingly under the QTcB prediction in both all-cause and cardiac mortality.

To the best of our knowledge, this is the first study to determine the best QT correction formula in an Iranian population. There are several reports in the literature regarding this comparison among formulas; however, they are generally based on hospitalized patients with no consideration given to a normal healthy population. Moreover, there is virtually no mortality rate for assessing and validating their interpretations. Furthermore, QTc intervals are clinically significant, especially with drug administration in apparently normal patients in society and for monitoring the side effects of many drugs, including psychiatry and cardiac medications. A great number of these people are not hospitalized, yet their QT intervals need to be considered before and during the administration of such drugs.

### Descriptive data of ECG parameters

CVD is the leading cause of premature death worldwide with 17.9 million deaths in 2012, which is expected to rise to 23 million deaths in 2030. Moreover, the low- and medium-income countries, especially the Eastern Mediterranean countries, account for 50 percent of deaths and 80% of the global burden from this disease [41]. The prevalence and prediction rate of CVD in Iran imply the importance of CVD [42]. ECG as a test with a high prediction value, can be used to predict the chance of CVD development [43]. Descriptive data showed that P duration, PR interval, QRS duration, P and QRS axis were higher in women. Heart rate and all QTc interval had a higher mean and SD in males than females. Pinto Filho et al. [44] in a large Brazilian population showed that mean of P duration, PR interval, QRS duration were higher in their male subjects which were in contrast to our results but in case the mean of P axis was in a line with our study. Moreover, the median of P duration, PR interval, P axis in the aforementioned study was higher than ours. The increasing trends of P duration, PR interval, QRS duration, P axis, and QTc intervals with increasing age were almost similar to other populations such as Brazilian [44], Netherlands [45], Indians [46], and Chinese [47].

### QT prolongation

Previous studies have reported that abnormal QT prolongation predisposes individuals to ventricular arrhythmias (such as VF, TdP, and SCD) [16, 18, 48]. It has also been suggested that QT interval prolongation may be a marker of subclinical atherosclerosis [49]. A qualitative review in 2004 [50] reported that there is no evidence of an increased risk of all-cause and cardiac mortality due to QTc prolongation. Although it has been reported that this risk may increase in patients with a history of CVD, a further meta-analysis study by Zhang et al. [51] suggested that there is a relative risk of more than 1.50 in the highest QT interval group compared to the lowest. Due to the importance of QT prolongation and a lack of studies on QT intervals, different QTc formulas, and their comparison in an Iranian population, the current study aimed to find the best QT correction formula and the normal limits based on mathematics and mortality.

### Comparing different QTc intervals

Several previous studies have compared different QTc formulas to determine which is the best. The most commonly used formula, Bazett’s, has been challenged in several studies [31, 52–56] due to reasons such as the over-correction of QTc intervals at higher heart rates and under-correction at lower heart rates. The current study also reports the same result. In Fig. 1, it is obvious that QTcB in short RR has much higher values of QTc interval and vice versa. Previous studies [31, 57] have suggested the use of QTcFri, QTcH, and QTcFra instead of QTcB. The current study also suggests these formulas as the best three based on mathematical methods. Luo et al. [31] reported that Bazett's formula is common simply because of its popularity among physicians and their students without considering previous studies. They have also demonstrated some QTc formulas are good for bradycardia while others are better for tachycardia. For example, the Framingham formula has less correction above 100 bpm, and the Hodges formula has less correction at heart rates below 60 bpm than others. Ultimately, they concluded that QTcH was the best formula for their population.

QTc and RR regression are accepted as an assessment for the accuracy of the correction formula [58]. In the ideal form, if the regression line has zero slopes, the value of QTc is statistically independent of RR. In a simpler explanation, the best correction formula is the one that minimizes the influence of RR. The positive and negative values of the slope are evidence for over- and under-correction of formulas. For example, in the positive slope of different QTcs between RR and QTc intervals, some over-correction is expected in RR intervals > 1.0 s, but some under-correction is expected in RR intervals < 1 s. The opposite is expected to happen in the favor of a negative correlation such as QTcB as discussed earlier (Table 4 and Fig. 1). With this evidence, it can be stated that QTcB was the poorest correction formula mathematically in the current study for both males and females. Moreover, the smallest slope and minimum influence of RR in QTc in males was related to QTcFri in this study.

There were also some differences in QTc intervals between males and females. Heart rates and all QTc intervals were statistically higher in females, which was expected and is in line with previous studies [52, 57]. Previous studies have also reported higher values of slope in women compared with men in QT interval and RR analyses [59]. The current study did not show the same in all of QTc intervals, but QTcFri and QTcFra slopes were higher in females. Moreover, the highest differences in slope between males and females belonged to QTcR. Interestingly, the only QTc interval which had a turning point (from a negative to a positive slope) between males and females was QTcFra (− 0.010 in males to 0.019 in females). For this gender difference, there are some possible mechanisms including differences in cardiac electrophysiology, sex hormones, and autonomic nervous system [60–63].

### Normal limits

The results of the current study showed that QTcB had the highest ULN among QTc formulas in both genders and the highest LLN in females. The highest LLN in male subjects was related to QTcR. The ULN and LLN of the best correction formula for the current study population, QTcFri, was near sex-specific clinical standards cutoffs, especially in males (450 ms for males and 470 ms for females [64]). There are also normal limits of differences in gender. Overall, the normal limits were higher in women. QTcB had a ULN at least 11 ms more than the other formulas and was the highest value among others which can be supported by previous studies [31, 57]. It is noteworthy that the abovementioned ULNs were driven from the population and, to some extent, the use of the upper domain of these QTc might be risky in cases of certain drug administration or drug studies. These ULNs should be investigated to be validated for clinical use.

### Mortality prediction

In the current study, QTcB, QTcFra, QTcR, and QTcD in all-cause mortality and QTcB in cardiac mortality had the highest sensitivity. Vandenberk et al. [57] recently reported that the highest sensitivity in 30-day mortality was related to QTcFri and QTcFra (27.9%) and in 1-year mortality was related to QTcFri (16.3%). It should be mentioned that the authors did not report the sensitivity of the QTc formulas in cardiac mortality or with a gender distinction. In the Cox regression, an increased risk of all-cause mortality was seen in prolonged QTcB, QTcFri, QTcFra, QTcR, and QTcD, and among them, the highest HR was related to QTcFra. In cardiac mortality, only prolonged QTcB showed an increased risk of mortality among others. It should be mentioned that these increased risks could not be observed in females. Previous studies of cases of mortality HR in prolonged QTc are limited and restricted to a few QTc formulas. The Framingham study [65] reported that in the age and sex-adjusted model, HR for CHD mortality was 1.14 (95% CI 1.10–1.18) in QTcB and 1.09 (95% CI 1.04–1.13) in QTcFra, which is in line with the current results regarding the superiority of QTcB in cardiac mortality compared to others. Schouten et al. [66] reported a 1.8 HR for CVD mortality in subjects with QTcB > 440 ms. Although these two studies had a much longer follow-up, they support the current results. Nielsen et al. [67] showed in a large population study that QTcFra ≥ 466 ms had an HR = 2.53 (95% CI, 2.15–2.98) in all-cause and an HR = 4.08 (95% CI, 2.93–5.69) in cardiac mortality in men. Unfortunately, these studies did not calculate HR in different QTc formulas for a better comparison. A recent study did compare HR in different QTc formulas [57] and reported that the highest HR in 1-year cardiac mortality was QTcR (5.64) followed by QTcB (4.48). Unfortunately, the researchers of that study focused on the all-cause mortality in the main text and concluded that QTcFri and QTcFra are the best correction formulas. In addition, their population was hospitalized and, consequently, had a higher mortality rate, which can be one reason for the differences between their findings and those of the current study.

### Limitations

Measuring QT interval, even by an automated method, may produce false-positive QT prolongation due to uncertain determination of QT intervals. Defining the T-wave termination may be challenging in some ECGs for both a physician's eye and computer algorithms [68]. Also, as a 10-s ECG was recorded there may be a problem with QT/RR hysteresis lag and although we tried to record ECG in the optimum state, the stability of heart rate may not be guaranteed [69].

The population approach for finding the best QTc formula may not be the right method, as we do not share the same QT/RR relationship. The individualized QTc formula should be the best method due to individual differences in people. This opinion has been also suggested by others [70, 71]. Unfortunately, a series of ECG for each participant was not available in this study, which makes this limitation an undeniable one.

The better performance of QTcB in mortality prediction may be due simply to over- and under-correction and its artificial prolongation of QTc intervals. This issue cannot be ignored, especially in the current population-based study.

The association of metabolic syndrome, hypertension, obesity, diabetes, impaired glucose tolerance, and elevated insulin levels [72–77] with prolonged QTc and the effect other factors discussed earlier [4–7, 10–14] on QT intervals and cardiovascular events should not be ignored. Moreover, there is an issue about the relation and interrelation of some of these factors on both QT intervals and cardiovascular events at the same time. Thus, the results of further studies should be adjusted with these factors to minimize their influence.

As another limitation, the observed results in males could not be exploited in females. Although the mortality rates of both genders were similar, no relationship could be shown between prolonged QTc and mortality in women. A longer follow-up study with a higher mortality rate and multi-variable adjustment by different analysis method such as machine learning or deep learning [78] should be conducted in the future.

Finally, an urban Iranian population may present different findings while our data were from rural regions. So the validation of the study's findings should be done externally with a different population to enable us to generalize the results of the current study.

## Conclusion

QT interval should be corrected by the heart rate of the individuals and there are several heart rate correction formulas. As the QTcFri had the smallest influence of RR in QTc in our study, it is suggested that QTcFri may be the best heart rate correction formula by focusing on mathematical methods. However, if we focus on cardiovascular deaths, despite its poorest performance in mathematical methods, QTcB may be the best one. Practically, it is suggested that physicians use QTcB for a better evaluation of the risks of cardiac mortality. However, in population-based studies, QTcFri might be the one to be used by researchers.

## Supplementary Information

**Additional file 1: Table S1.** Descriptive data of electrocardiogram parameters stratified by age and sex. **Table S2.** Mean difference and correlation between different QTc formulas. **Table S3.** Results of Bland-Altman analysis between different QTc formulas. **Figure F1.** Bland-Altman graphs of the difference between two QTc intervals versus average. **Table S4.** Sensitivity Analysis of All-cause and cardiac mortality prediction by QTc > ULN in different QTc intervals in female. **Table S5.** Cox regression analysis of unadjusted model of All-cause and cardiac mortality prediction by QTc > ULN in different QTc intervals in female. **Table S6.** Cox regression analysis of the un- and multivariate-adjusted model of All-cause and cardiac mortality prediction by QTc > ULN in different QTc intervals in both genders. **Table S7.** The detailed result of cox regression analysis of the uni- and multivariate-adjusted model of All-cause and cardiac mortality prediction by QTc > ULN in different QTc intervals in males. **Table S8.** The detailed result of cox regression analysis multivariate-adjusted model of All-cause and cardiac mortality prediction by QTc > ULN in different QTc intervals in males.

## Data Availability

The datasets used and/or analyzed during the current study are available from the corresponding author on reasonable request to the corresponding author.

## Abbreviations

ECG: Electrocardiogram
LQTS: Long QT syndrome
VF: Ventricular fibrillation
TdP: Torsade de Pointes
SCD: Sudden cardiac death
QTc: Corrected QT interval
QTcB: Corrected QT interval by Bazett’s formula
QTcFri: Corrected QT interval by Fridericia’s formula
QTcD: Corrected QT interval by Dmitrienko’s formula
QTcFra: Corrected QT interval by Framingham’s formula
QTcH: Corrected QT interval by Hodges’s formula
QTcR: Corrected QT interval by Rautaharju’s formula
CVD: Cardiovascular diseases
CHD: Coronary heart disease
MI: Myocardial infarction
ULN: Upper limits of normal
LLN: Lower limits of normal
PPV: Positive predictive value
NPV: Negative predictive value
HR: Hazard ratio
