# Supplementary material for: The best QT correction formula in a non-hospitalized population: the Fasa PERSIAN cohort study
Source: BMC Cardiovasc Disord. 2022 Feb 16;22:52. doi: 10.1186/s12872-022-02502-2 (PMC8851728; doi:10.1186/s12872-022-02502-2)
Supplement: Supplementary file 1 — Additional file 1: Table S1. Descriptive data of electrocardiogram parameters stratified by age and sex. Table S2. Mean difference and correlation between different QTc formulas. Table S3. Results of Bland-Altman analysis between different QTc formulas. Figure F1. Bland-Altman graphs of the difference between two QTc intervals versus average. Table S4. Sensitivity Analysis of All-cause and cardiac mortality prediction by QTc > ULN in different QTc intervals in female. Table S5. Cox regression analysis of unadjusted model of All-cause and cardiac mortality prediction by QTc > ULN in different QTc intervals in female. Table S6. Cox regression analysis of the un- and multivariate-adjusted model of All-cause and cardiac mortality prediction by QTc > ULN in different QTc intervals in both genders. Table S7. The detailed result of cox regression analysis of the uni- and multivariate-adjusted model of All-cause and cardiac mortality prediction by QTc > ULN in different QTc intervals in males. Table S8. The detailed result of cox regression analysis multivariate-adjusted model of All-cause and cardiac mortality prediction by QTc > ULN in different QTc intervals in males. [file 12872_2022_2502_MOESM1_ESM.docx]

**Supplementary Material**

**Table S1. Descriptive data of electrocardiogram parameters stratified by age and sex**

|  | | Male | | | | | | | | | | | | Female | | | | | | | | | | | |  |
| --- | --- | --- | --- | --- | --- | --- | --- | --- | --- | --- | --- | --- | --- | --- | --- | --- | --- | --- | --- | --- | --- | --- | --- | --- | --- | --- |
|  |  | <50 years  (n = 2253) | | | | ≥50 years  (n = 1740) | | | | Total  (n = 3993) | | | | <50 years  (n = 1759) | | | | ≥50 years  (n = 1319) | | | | Total  (n = 3078) | | | |  |
|  |  | Mean | | SD | | Mean | | SD | | Mean | | SD | | Mean | | SD | | Mean | | SD | | Mean | | SD | |  |
| Hear rate (bpm) | | | 75.9 | | 11.3 | | 74.3 | | 11.9 | | 75.2 | | 11.6 | | 65.6 | | 10.7 | | 66.6 | | 11.1 | | 66.0 | | 10.9 | |
| P duration (ms) | | | 97.9 | | 22.1 | | 102.1 | | 22.6 | | 99.7 | | 22.4 | | 98.3 | | 25.9 | | 102.5 | | 25.9 | | 100.1 | | 26.0 | |
| PR interval (ms) | | | 134.1 | | 31.2 | | 140.7 | | 32.5 | | 136.9 | | 32.0 | | 138.6 | | 36.4 | | 143.2 | | 36.9 | | 140.6 | | 36.7 | |
| QRS duration (ms) | | | 94.3 | | 9.1 | | 97.0 | | 10.7 | | 95.5 | | 9.9 | | 98.4 | | 10.0 | | 98.7 | | 11.6 | | 98.5 | | 10.7 | |
| P axis (°) | | | 43.1 | | 23.1 | | 46.9 | | 22.0 | | 44.8 | | 22.7 | | 47.2 | | 26.6 | | 53.1 | | 24.4 | | 49.7 | | 25.8 | |
| QRS axis (°) | | | 35.7 | | 30.9 | | 28.1 | | 32.5 | | 32.4 | | 31.8 | | 43.7 | | 42.0 | | 31.8 | | 43.8 | | 38.6 | | 43.2 | |
| QT interval (ms) | | | 391.7 | | 34.7 | | 400.8 | | 39.6 | | 395.6 | | 37.2 | | 401.6 | | 36.2 | | 406.7 | | 36.3 | | 403.8 | | 36.3 | |
| RR (s) | | | 0.81 | | 0.12 | | 0.83 | | 0.13 | | 0.82 | | 0.12 | | 0.94 | | 0.15 | | 0.93 | | 0.15 | | 0.93 | | 0.15 | |
| QTc(ms) | Bazett | | 437.6 | | 32.8 | | 442.3 | | 34.4 | | 439.6 | | 33.6 | | 416.7 | | 31.6 | | 425.0 | | 31.8 | | 420.2 | | 31.9 | |
|  | Fridericia | | 421.4 | | 30.4 | | 427.7 | | 33.0 | | 424.2 | | 31.7 | | 411.3 | | 29.4 | | 418.5 | | 29.3 | | 414.4 | | 29.6 | |
|  | Framingham | | 421.2 | | 29.1 | | 427.2 | | 31.7 | | 423.8 | | 30.4 | | 411.1 | | 29.0 | | 418.1 | | 28.8 | | 414.1 | | 29.1 | |
|  | Hodges | | 419.4 | | 28.4 | | 425.7 | | 31.4 | | 422.2 | | 29.9 | | 411.4 | | 29.4 | | 418.2 | | 28.8 | | 414.3 | | 29.3 | |
|  | Dmitrienko | | 429.0 | | 31.1 | | 434.6 | | 33.2 | | 431.5 | | 32.2 | | 413.8 | | 29.9 | | 421.6 | | 29.9 | | 417.1 | | 30.2 | |
|  | Rautaharju | | 427.2 | | 29.2 | | 432.6 | | 31.4 | | 429.5 | | 30.3 | | 419.0 | | 29.6 | | 426.4 | | 29.4 | | 422.2 | | 29.8 | |
| QT/QTc | Bazett | | 0.897 | | 0.066 | | 0.907 | | 0.071 | | 0.901 | | 0.068 | | 0.966 | | 0.077 | | 0.959 | | 0.079 | | 0.963 | | 0.078 | |
|  | Fridericia | | 0.929 | | 0.045 | | 0.937 | | 0.049 | | 0.932 | | 0.047 | | 0.976 | | 0.052 | | 0.972 | | 0.053 | | 0.974 | | 0.052 | |
|  | Framingham | | 0.930 | | 0.049 | | 0.937 | | 0.049 | | 0.933 | | 0.049 | | 0.977 | | 0.056 | | 0.973 | | 0.058 | | 0.975 | | 0.057 | |
|  | Hodges | | 0.934 | | 0.048 | | 0.941 | | 0.049 | | 0.937 | | 0.049 | | 0.976 | | 0.045 | | 0.972 | | 0.047 | | 0.974 | | 0.046 | |
|  | Dmitrienko | | 0.913 | | 0.055 | | 0.922 | | 0.060 | | 0.917 | | 0.057 | | 0.971 | | 0.064 | | 0.966 | | 0.066 | | 0.969 | | 0.064 | |
|  | Rautaharju | | 0.917 | | 0.060 | | 0.926 | | 0.057 | | 0.921 | | 0.059 | | 0.959 | | 0.066 | | 0.955 | | 0.069 | | 0.957 | | 0.068 | |

QTc: Corrected QT interval, SD: Standard deviation

**Table S2-a. Mean difference and correlation between different QTc formulas in male**

| **QTc intervals** | **Mean (ms)** | **95% CI (ms)** | **P-value*** | **Pearson Correlation** | **P-value**** |
| --- | --- | --- | --- | --- | --- |
| Bazett – Fridericia | 5.84 | 5.44 to 6.24 | **<0.001** | 0.935 | **<0.001** |
| Bazett - Framingham | 6.15 | 5.78 to 6.52 | **<0.001** | 0.945 | **<0.001** |
| Bazett - Hodges | 5.94 | 5.42 to 6.46 | **<0.001** | 0.889 | **<0.001** |
| Bazett - Rautahatju | -1.91 | -2.14 to -1.67 | **<0.001** | 0.980 | **<0.001** |
| Bazett - Dmitrienko | 3.10 | 2.88 to 3.31 | **<0.001** | 0.983 | **<0.001** |
| Fridericia - Framingham | 0.30 | 0.20 to 0.41 | **<0.001** | 0.995 | **<0.001** |
| Fridericia - Hodges | 0.10 | -0.06 to 0.26 | 0.234 | 0.987 | **<0.001** |
| Fridericia - Rautahatju | -7.75 | -2.14 to -1.67 | **<0.001** | 0.973 | **<0.001** |
| Fridericia - Dmitrienko | -2.74 | -2.93 to -2.55 | **<0.001** | 0.984 | **<0.001** |
| Framingham - Hodges | -0.20 | -2.14 to -1.67 | 0.094 | 0.972 | **<0.001** |
| Framingham - Rautahatju | -8.06 | -2.14 to -1.67 | **<0.001** | 0.988 | **<0.001** |
| Framingham - Dmitrienko | -3.055 | -3.23 to -2.87 | **<0.001** | 0.987 | **<0.001** |
| Hodges - Rautahatju | -7.85 | -2.14 to -1.67 | **<0.001** | 0.935 | **<0.001** |
| Hodges - Dmitrienko | -2.84 | -3.16 to -2.52 | **<0.001** | 0.954 | **<0.001** |
| Rautahatju - Dmitrienko | 5.01 | 4.88 to 5.13 | **<0.001** | 0.993 | **<0.001** |

**Table S2-b. Mean differences and correlations between different QTc formulas in female**

| **QTc intervals** | **Mean (ms)** | **95% CI (ms)** | **P-value*** | **Pearson Correlation** | **P-value**** |
| --- | --- | --- | --- | --- | --- |
| Bazett - Fridericia | 15.58 | 15.16 to 15.99 | **<0.001** | 0.918 | **<0.001** |
| Bazett - Framingham | 15.80 | 15.42 to 16.17 | **<0.001** | 0.932 | **<0.001** |
| Bazett - Hodges | 17.47 | 17.11 to 17.82 | **<0.001** | 0.942 | **<0.001** |
| Bazett - Rautahatju | 10.22 | 9.88 to 10.56 | **<0.001** | 0.947 | **<0.001** |
| Bazett - Dmitrienko | 8.17 | 7.98 to 8.35 | **<0.001** | 0.985 | **<0.001** |
| Fridericia - Framingham | 0.22 | -0.04 to 0.48 | 0.103 | 0.966 | **<0.001** |
| Fridericia - Hodges | 1.89 | 1.61 to 2.17 | **<0.001** | 0.962 | **<0.001** |
| Fridericia - Rautahatju | -5.35 | -5.70 to -5.00 | **<0.001** | 0.939 | **<0.001** |
| Fridericia - Dmitrienko | -7.28 | -7.44 to -7.12 | **<0.001** | 0.987 | **<0.001** |
| Framingham - Hodges | 1.66 | 1.50 to 1.83 | **<0.001** | 0.985 | **<0.001** |
| Framingham - Rautahatju | -5.57 | -5.80 to -5.34 | **<0.001** | 0.971 | **<0.001** |
| Framingham - Dmitrienko | -7.63 | -7.83 to -7.42 | **<0.001** | 0.979 | **<0.001** |
| Hodges - Rautahatju | -7.24 | -7.52 to -6.96 | **<0.001** | 0.985 | **<0.001** |
| Hodges - Dmitrienko | -9.30 | -9.49 to -9.10 | **<0.001** | 0.981 | **<0.001** |
| Rautahatju - Dmitrienko | -1.95 | -2.08 to -1.82 | **<0.001** | 0.992 | **<0.001** |

95% CI= 95% Confidence Interval, ms= milliseconds, P-value reported as the result of * Paired t-test ** Pearson Correlation analysis. Statistically significant P-values are bolded(P-value<0.05).

**Table S3-a. Results of Bland-Altman analysis between different QTc formulas in male**

| **QTc intervals** | **Bias (ms)** | **SD (ms)** | **95% limits of agreement (ms)** |
| --- | --- | --- | --- |
| Bazett - Fridericia | 5.84 | 11.34 | -16.39 to 28.09 |
| Bazett - Framingham | 6.15 | 10.50 | -14.42 to 26.73 |
| Bazett - Hodges | 5.94 | 14.65 | -22.77 to 34.66 |
| Bazett - Rautahatju | -1.91 | 6.564 | -14.77 to 10.95 |
| Bazett - Dmitrienko | 3.10 | 5.970 | -8.602 to 14.80 |
| Fridericia - Framingham | 0.30 | 3.048 | -5.66 to 6.28 |
| Fridericia - Hodges | 0.10 | 4.685 | -9.08 to 9.28 |
| Fridericia - Rautahatju | -7.75 | 6.841 | -21.16 to 5.65 |
| Fridericia - Dmitrienko | -2.74 | 5.379 | -13.29 to 7.79 |
| Framingham - Hodges | -0.20 | 6.869 | -13.67 to13.25 |
| Framingham - Rautahatju | -8.06 | 4.679 | -17.23 to 1.10 |
| Framingham - Dmitrienko | -3.05 | 4.978 | -12.81 to 6.70 |
| Hodges - Rautahatju | -7.85 | 10.69 | -28.82 to 13.10 |
| Hodges - Dmitrienko | -2.84 | 9.033 | -20.55 to 14.85 |
| Rautahatju - Dmitrienko | 5.01 | 3.550 | -1.947 to 11.97 |

SD=Standard Deviation, ms= milliseconds, lower and upper limits of agreement was calculated by “-1.96*SD + Bias” and “+1.96*SD + Bias” respectively.

| **QTc intervals** | **Bias (ms)** | **SD (ms)** | **95% limits of agreement (ms)** |
| --- | --- | --- | --- |
| Bazett - Fridericia | 15.45 | 10.97 | -6.059 to 36.96 |
| Bazett - Framingham | 15.80 | 12.18 | -8.080 to 39.68 |
| Bazett - Hodges | 17.47 | 11.42 | -4.916 to 39.857 |
| Bazett - Rautahatju | 10.12 | 8.84 | -7.218 to 27.46 |
| Bazett - Dmitrienko | 8.17 | 5.83 | -3.260 to 19.60 |
| Fridericia - Framingham | 0.34 | 3.12 | -5.785 to 6.48 |
| Fridericia - Hodges | 2.01 | 4.22 | -6.273 to 10.30 |
| Fridericia - Rautahatju | -5.32 | 4.78 | -14.69 to 4.03 |
| Fridericia - Dmitrienko | -7.28 | 5.14 | -17.36 to 2.79 |
| Framingham - Hodges | 1.66 | 5.21 | -8.559 to 11.89 |
| Framingham - Rautahatju | -5.67 | 3.86 | -13.25 to 1.90 |
| Framingham - Dmitrienko | -7.63 | 6.68 | -20.72 to 5.46 |
| Hodges - Rautahatju | -7.34 | 6.25 | -19.59 to 4.90 |
| Hodges - Dmitrienko | -9.30 | 6.40 | -21.85 to 3.25 |
| Rautahatju - Dmitrienko | -1.95 | 4.31 | -10.41 to 6.50 |

**Table S3-b. Results of Bland-Altman analysis between different QTc formulas in female**

SD=Standard Deviation, ms= milliseconds, lower and upper limits of agreement was calculated by “-1.96*SD + Bias” and “+1.96*SD + Bias” respectively.

**
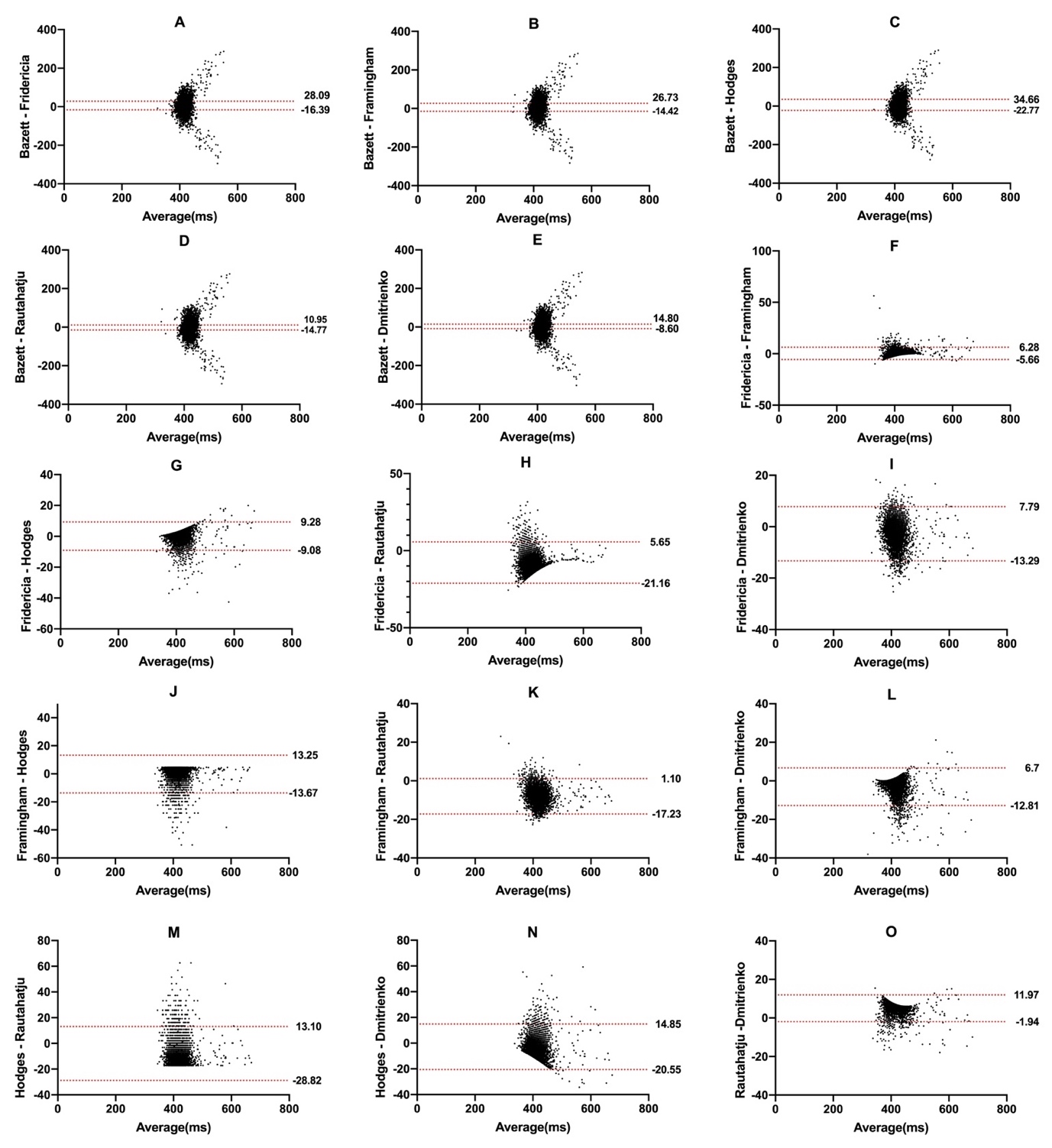
 Figure S1-a. Bland-Altman graphs of the difference between two QTc intervals versus average in male.** QTc= Corrected QT interval, A. Bland–Altman analysis for comparison of Bazett and Fridericia, B. Bland–Altman analysis for comparison of Bazett and Framingham C. Bland–Altman analysis for comparison of Bazett and Hodges D. Bland–Altman analysis for comparison of Bazett and Rautahatju E. Bland–Altman analysis for comparison of Bazett and Dmitrienko F. Bland–Altman analysis for comparison of Fridericia and Framingham G. Bland–Altman analysis for comparison of Fridericia and Hodges H. Bland–Altman analysis for comparison of Fridericia and Rautahatju I. Bland–Altman analysis for comparison of Fridericia and Dmitrienko J. Bland–Altman analysis for comparison of Framingham and Hodges K. Bland–Altman analysis for comparison of Framingham and Rautahatju L. Bland–Altman analysis for comparison of Framingham and Dmitrienko M. Bland–Altman analysis for comparison of Hodges and Rautahatju N. Bland–Altman analysis for comparison of Hodges and Dmitrienko O. Bland–Altman analysis for comparison of Rautahatju and Dmitrienko. The upper and lower redlines are respectively upper and lower agreements levels.

**
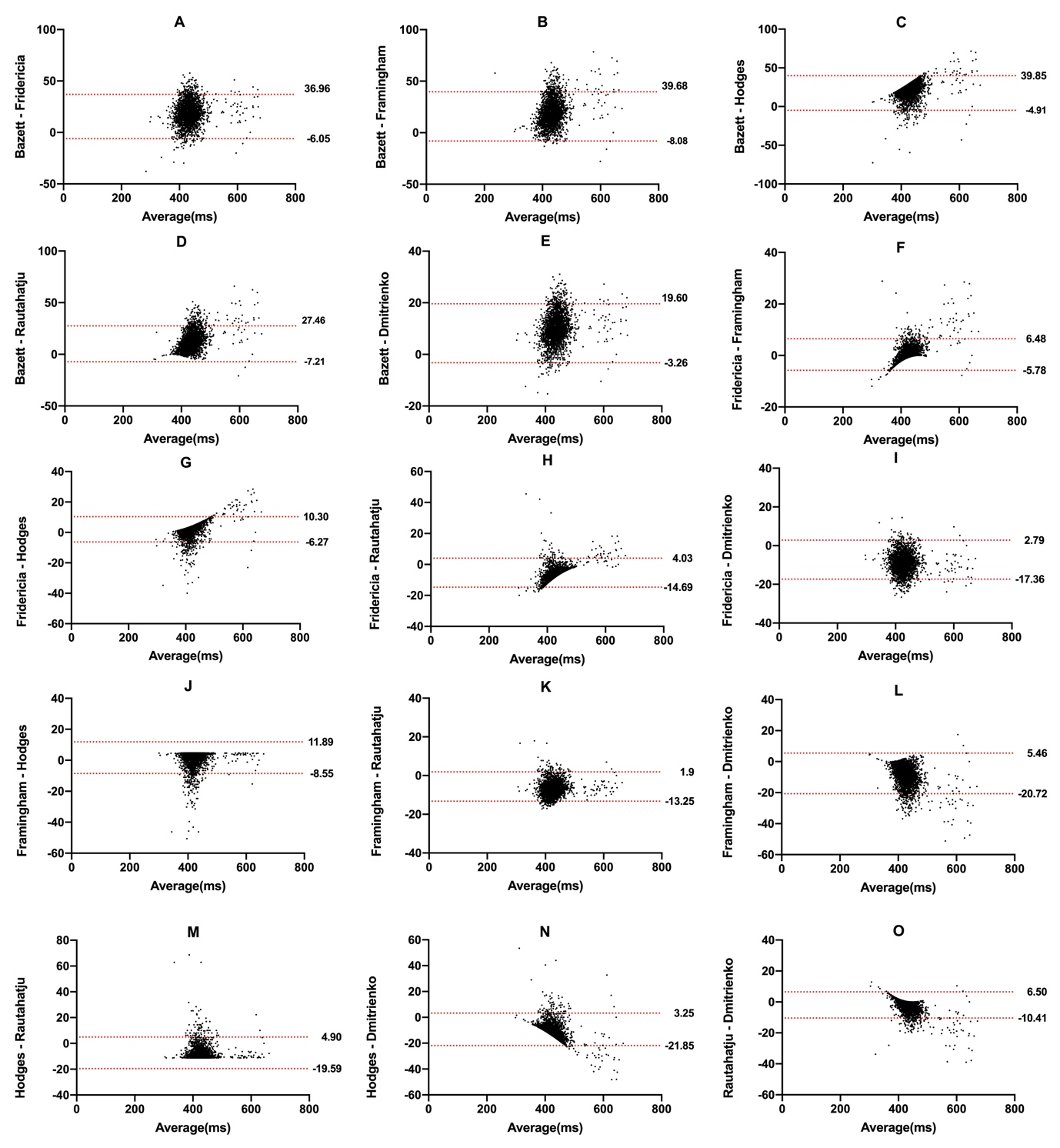
Figure S1-b. Bland-Altman graphs of the difference between two QTc intervals in average in female.** QTc= Corrected QT interval A. Bland–Altman analysis for comparison of Bazett and Fridericia, B. Bland–Altman analysis for comparison of Bazett and Framingham C. Bland–Altman analysis for comparison of Bazett and Hodges D. Bland–Altman analysis for comparison of Bazett and Rautahatju E. Bland–Altman analysis for comparison of Bazett and Dmitrienko F. Bland–Altman analysis for comparison of Fridericia and Framingham G. Bland–Altman analysis for comparison of Fridericia and Hodges H. Bland–Altman analysis for comparison of Fridericia and Rautahatju I. Bland–Altman analysis for comparison of Fridericia and Dmitrienko J. Bland–Altman analysis for comparison of Framingham and Hodges K. Bland–Altman analysis for comparison of Framingham and Rautahatju L. Bland–Altman analysis for comparison of Framingham and Dmitrienko M. Bland–Altman analysis for comparison of Hodges and Rautahatju N. Bland–Altman analysis for comparison of Hodges and Dmitrienko O. Bland–Altman analysis for comparison of Rautahatju and Dmitrienko. The upper and lower redlines are respectively upper and lower agreements levels.

**Table S4. Sensitivity Analysis of All-cause and cardiac mortality prediction by QTc>ULN in different QTc intervals in female.**

| **QTc intervals** | **All-cause mortality** | **Cardiac mortality** | | | | **QTc intervals** | | | |
| --- | --- | --- | --- | --- | --- | --- | --- | --- | --- |
|  | QTc >ULN | Sens (%) | Spec (%) | NPV (%) | PPV (%) | Sens (%) | Spec (%) | NPV (%) | PPV (%) |
| QTcB | 6.8% | 3.03 | 93.20 | 99.14 | 0.37 | 0.00 | 93.20 | 99.48 | 0.00 |
| QTcFri | 7% | 0.00 | 92.95 | 99.11 | 0.00 | 0.00 | 92.79 | 99.48 | 0.00 |
| QTcFra | 6.9% | 0.00 | 93.08 | 99.11 | 0.00 | 0.00 | 93.10 | 99.48 | 0.00 |
| QTcH | 7.2% | 3.03 | 92.77 | 99.13 | 0.34 | 5.26 | 92.80 | 99.51 | 0.34 |
| QTcR | 6.9% | 0.00 | 93.00 | 99.11 | 0.00 | 0.00 | 92.88 | 99.47 | 0.00 |
| QTcD | 7.0% | 0.00 | 92.92 | 99.11 | 0.00 | 0.00 | 92.95 | 99.48 | 0.00 |

ULN=upper limit of normal, QTc= Corrected QT interval, Sens= Sensitivity, Spec= Specificity, PPV= Positive Predictive Value, and NPV=Negative Predictive Value, Bazett’s correction formula (QTcB), Fridericia’s correction formula (QTcFri), Dmitrienko’s correction formula (QTcD), Framingham’s correction formula (QTcFra), Hodges’s correction formula (QTcH), Rautaharju’s correction formula (QTcR).

**Table S5. Cox regression analysis of unadjusted model of All-cause and cardiac mortality prediction by QTc>ULN in different QTc intervals in female.**

|  | **All-cause mortality** | | | **Cardiac mortality** | | |
| --- | --- | --- | --- | --- | --- | --- |
|  | **HR** | **95%CI** | **P-VALUE** | **HR** | **95%CI** | **P-VALUE** |
| QTcB | 0.437 | 0.60 to 3.19 | 0.415 | 0.045 | 0.00 to 127.26 | 0.444 |
| QTcFri | 0.045 | 0.00 to 17.09 | 0.306 | 0.045 | 0.00 to 111.39 | 0.436 |
| QTcFra | 0.045 | 0.00 to 18.13 | 0.311 | 0.045 | 0.00 to 119.51 | 0.440 |
| QTcH | 0.410 | 0.056 to 3.00 | 0.380 | 0.725 | 0.09 to 5.43 | 0.754 |
| QTcR | 0.045 | 0.00 to 17.46 | 0.307 | 0.045 | 0.00 to 116.36 | 0.439 |
| QTcD | 0.045 | 0.00 to 16.84 | 0.304 | 0.045 | 0.00 to 109.84 | 0.435 |

ULN=upper limit of normal, HR= Hazard Ratio, CI= confidence interval, QTc= Corrected QT interval, Sens= Sensitivity, Spec= Specificity, PPV= Positive Predictive Value, and NPV=Negative Predictive Value, Bazett’s correction formula (QTcB), Fridericia’s correction formula (QTcFri), Dmitrienko’s correction formula (QTcD), Framingham’s correction formula (QTcFra), Hodges’s correction formula (QTcH), Rautaharju’s correction formula (QTcR).

**Table S6. Cox regression analysis of the un- and multivariate-adjusted model of All-cause and cardiac mortality prediction by QTc>ULN in different QTc intervals in both genders.**

|  | **Unadjusted** | | | | | | **Multivariate-adjusted** | | | | | | |
| --- | --- | --- | --- | --- | --- | --- | --- | --- | --- | --- | --- | --- | --- |
|  | **All-cause mortality** | | | **Cardiac mortality** | | | **All-cause mortality** | | | **Cardiac mortality** | | | |
|  | HR | 95%CI | P-VALUE | HR | 95%CI | P-VALUE | HR | 95%CI | P-VALUE | HR | 95%CI | P-VALUE |  |
| QTcB | 1.82 | 0.91 to 3.64 | 0.092 | 2.17 | 0.92 to 5.13 | 0.077 | 1.236 | 0.61 to 2.52 | 0.560 | 1.297 | 0.54 to 3.14 | 0.564 |  |
| QTcFri | 1.36 | 0.62 to 2.95 | 0.440 | 1.36 | 0.49 to 3.80 | 0.556 | 1.163 | 0.53 to 2.54 | 0.705 | 1.081 | 0.38 to 3.03 | 0.883 |  |
| QTcFra | 1.62 | 0.78 to 3.36 | 0.197 | 1.40 | 0.50 to 3.91 | 0.522 | 1.417 | 0.68 to 2.96 | 0.354 | 1.145 | 0.41 to 3.22 | 0.797 |  |
| QTcH | 1.14 | 0.50 to 2.63 | 0.753 | 1.36 | 0.49 to 3.79 | 0.560 | 1.031 | 0.44 to 2.39 | 0.942 | 1.148 | 0.41 to 3.24 | 0.795 |  |
| QTcR | 1.59 | 0.77 to 3.31 | 0.212 | 1.38 | 0.49 to 3.85 | 0.540 | 1.31 | 0.63 to 2.73 | 0.471 | 1.041 | 0.37 to 2.92 | 0.939 |  |
| QTcD | 1.57 | 0.75 to 3.25 | 0.229 | 1.36 | 0.49 to 3.78 | 0.562 | 1.231 | 0.59 to 2.56 | 0.578 | 0.974 | 0.35 to 2.73 | 0.961 |  |

ULN=upper limit of normal, HR= Hazard Ratio, CI= confidence interval, statistically significant HRs are bolded. QTc= Corrected QT interval, Bazett’s correction formula (QTcB), Fridericia’s correction formula (QTcFri), Dmitrienko’s correction formula (QTcD), Framingham’s correction formula (QTcFra), Hodges’s correction formula (QTcH), Rautaharju’s correction formula (QTcR). Multivariate-adjust was done with age and heart and gender

**Table S7. The detailed result of cox regression analysis of the uni- and multivariate-adjusted model of All-cause and cardiac mortality prediction by QTc>ULN in different QTc intervals in males**

|  | | **All-cause mortality** | | | | **Cardiac mortality** | | | |
| --- | --- | --- | --- | --- | --- | --- | --- | --- | --- |
|  |  | P-value | HR | 95.0% CI | | P-value | HR | 95.0% CI | |
| Bazett | Unadjusted |  |  |  |  |  |  |  |  |
|  | QTc | **0.004** | **3.029** | **1.413** | **6.492** | **0.002** | **4.317** | **1.733** | **10.749** |
|  | Adjusted |  |  |  |  |  |  |  |  |
|  | QTc | **0.036** | **2.360** | **1.056** | **5.276** | **0.032** | **2.917** | **1.093** | **7.780** |
|  | Heart Rate | 0.273 | 1.014 | 0.989 | 1.039 | 0.270 | 1.018 | 0.986 | 1.051 |
|  | Age | 0.002 | 1.051 | 1.019 | 1.084 | <0.001 | 1.095 | 1.047 | 1.145 |
| Fridericia | Unadjusted |  |  |  |  |  |  |  |  |
|  | QTc | **0.018** | **2.637** | **1.180** | **5.896** | 0.070 | 2.674 | 0.922 | 7.761 |
|  | Adjusted |  |  |  |  |  |  |  |  |
|  | QTc | **0.025** | **2.521** | **1.126** | **5.644** | 0.096 | 2.475 | 0.851 | 7.192 |
|  | Heart Rate | 0.087 | 1.021 | 0.997 | 1.046 | 0.068 | 1.028 | 0.998 | 1.060 |
|  | Age | 0.001 | 1.052 | 1.020 | 1.085 | <0.001 | 1.098 | 1.050 | 1.148 |
| Framingham | Unadjusted |  |  |  |  |  |  |  |  |
|  | QTc | **0.003** | **3.208** | **1.496** | **6.876** | 0.060 | 2.774 | 0.956 | 8.051 |
|  | Adjusted |  |  |  |  |  |  |  |  |
|  | QTc | **0.004** | **3.079** | **1.432** | **6.620** | 0.081 | 2.589 | 0.889 | 7.536 |
|  | Heart Rate | 0.076 | 1.022 | 0.998 | 1.047 | 0.063 | 1.029 | 0.998 | 1.061 |
|  | Age | 0.002 | 1.051 | 1.019 | 1.085 | <0.001 | 1.098 | 1.049 | 1.148 |
| Hodges | Unadjusted |  |  |  |  |  |  |  |  |
|  | QTc | 0.195 | 1.848 | 0.730 | 4.676 | 0.266 | 1.979 | 0.594 | 6.591 |
|  | Adjusted |  |  |  |  |  |  |  |  |
|  | QTc | 0.171 | 1.921 | 0.754 | 4.898 | 0.236 | 2.081 | 0.620 | 6.987 |
|  | Heart Rate | 0.072 | 1.022 | 0.998 | 1.047 | 0.057 | 1.029 | 0.999 | 1.061 |
|  | Age | 0.001 | 1.053 | 1.021 | 1.086 | <0.001 | 1.099 | 1.050 | 1.149 |
| Rautaharju | Unadjusted |  |  |  |  |  |  |  |  |
|  | QTc | **0.003** | **3.132** | **1.461** | **6.714** | 0.067 | 2.708 | 0.933 | 7.858 |
|  | Adjusted |  |  |  |  |  |  |  |  |
|  | QTc | **0.007** | **2.839** | **1.323** | **6.095** | 0.122 | 2.319 | 0.798 | 6.739 |
|  | Heart Rate | 0.114 | 1.020 | 0.995 | 1.045 | 0.082 | 1.027 | 0.997 | 1.059 |
|  | Age | 0.001 | 1.052 | 1.020 | 1.085 | <0.001 | 1.098 | 1.050 | 1.148 |
| Dmitrienko | Unadjusted |  |  |  |  |  |  |  |  |
|  | QTc | **0.004** | **3.080** | **1.437** | **6.602** | 0.072 | 2.662 | 0.917 | 7.725 |
|  | Adjusted |  |  |  |  |  |  |  |  |
|  | QTc | **0.013** | **2.651** | **1.229** | **5.719** | 0.179 | 2.087 | 0.714 | 6.104 |
|  | Heart Rate | 0.150 | 1.018 | 0.994 | 1.043 | 0.098 | 1.026 | 0.995 | 1.058 |
|  | Age | 0.001 | 1.051 | 1.019 | 1.085 | <0.001 | 1.098 | 1.049 | 1.148 |

QTc= Corrected QT interval, ULN=upper limit of normal, HR= Hazard Ratio, CI= confidence interval, statistically significant HRs are bolded.

**Table S8. The detailed result of cox regression analysis multivariate-adjusted model of All-cause and cardiac mortality prediction by QTc>ULN in different QTc intervals in males**

|  | **All-cause mortality** | | | | | **Cardiac mortality** | | | | | |
| --- | --- | --- | --- | --- | --- | --- | --- | --- | --- | --- | --- |
|  | Variables | P-value | HR | 95.0% CI | | Variables | P-value | HR | 95.0% CI | | |
| Step 1 | Heart Rate | 0.200 | 1.018 | 0.991 | 1.045 | Heart Rate | 0.350 | 1.017 | | 0.982 | 1.053 |
|  | Age | 0.002 | 1.050 | 1.018 | 1.084 | Age | <0.001 | 1.094 | | 1.046 | 1.144 |
|  | QTcB | 0.561 | 1.564 | 0.346 | 7.082 | QTcB | 0.078 | 4.293 | | 0.851 | 21.660 |
|  | QTcFri | 0.264 | 0.245 | 0.021 | 2.898 | QTcFri | 0.995 | 1.015 | | 0.006 | 165.276 |
|  | QTcFra | 0.044 | 13.502 | 1.075 | 169.528 | QTcFra | 0.562 | 4.013 | | 0.037 | 437.279 |
|  | QTcH | 0.278 | 0.418 | 0.086 | 2.021 | QTcH | 0.566 | 0.532 | | 0.061 | 4.598 |
|  | QTcR | 0.856 | 1.302 | 0.074 | 22.764 | QTcR | 0.907 | 1.293 | | 0.017 | 97.226 |
|  | QTcD | 0.924 | 0.886 | 0.074 | 10.546 | QTcD | 0.421 | 0.222 | | 0.006 | 8.662 |
| Step 2 | Heart Rate | 0.199 | 1.018 | 0.991 | 1.045 | Heart Rate | 0.350 | 1.017 | | 0.982 | 1.053 |
|  | Age | 0.002 | 1.050 | 1.018 | 1.084 | Age | <0.001 | 1.094 | | 1.046 | 1.144 |
|  | QTcB | 0.551 | 1.516 | 0.386 | 5.960 | QTcB | 0.077 | 4.294 | | 0.853 | 21.627 |
|  | QTcFri | 0.264 | 0.245 | 0.021 | 2.891 | QTcFra | 0.386 | 4.053 | | 0.171 | 95.899 |
|  | QTcFra | 0.044 | 13.449 | 1.069 | 169.128 | QTcH | 0.557 | 0.532 | | 0.065 | 4.369 |
|  | QTcH | 0.279 | 0.419 | 0.087 | 2.024 | QTcR | 0.905 | 1.296 | | 0.019 | 89.650 |
|  | QTcR | 0.877 | 1.194 | 0.126 | 11.313 | QTcD | 0.419 | 0.222 | | 0.006 | 8.503 |
| Step 3 | Heart Rate | 0.201 | 1.018 | 0.991 | 1.045 | Heart Rate | 0.351 | 1.017 | | 0.982 | 1.053 |
|  | Age | 0.002 | 1.050 | 1.018 | 1.083 | Age | <0.001 | 1.094 | | 1.046 | 1.144 |
|  | QTcB | 0.480 | 1.578 | 0.445 | 5.600 | QTcB | 0.077 | 4.301 | | 0.854 | 21.658 |
|  | QTcFri | 0.270 | 0.248 | 0.021 | 2.954 | QTcFra | 0.275 | 4.484 | | 0.303 | 66.392 |
|  | QTcFra | 0.005 | 15.333 | 2.299 | 102.274 | QTcH | 0.555 | 0.530 | | 0.065 | 4.352 |
|  | QTcH | 0.272 | 0.415 | 0.086 | 1.993 | QTcD | 0.299 | 0.261 | | 0.021 | 3.306 |
| Step 4 | Heart Rate | 0.098 | 1.021 | 0.996 | 1.047 | Heart Rate | 0.315 | 1.018 | | 0.983 | 1.053 |
|  | Age | 0.002 | 1.051 | 1.019 | 1.084 | Age | <0.001 | 1.095 | | 1.047 | 1.145 |
|  | QTcFri | 0.367 | 0.353 | 0.037 | 3.398 | QTcB | 0.085 | 4.164 | | 0.823 | 21.060 |
|  | QTcFra | 0.005 | 15.151 | 2.272 | 101.023 | QTcFra | 0.371 | 2.744 | | 0.300 | 25.084 |
|  | QTcH | 0.283 | 0.423 | 0.088 | 2.035 | QTcD | 0.308 | 0.268 | | 0.021 | 3.370 |
| Step 5 | Heart Rate | 0.111 | 1.020 | 0.995 | 1.046 | Heart Rate | 0.411 | 1.014 | | 0.981 | 1.049 |
|  | Age | 0.002 | 1.051 | 1.019 | 1.084 | Age | <0.001 | 1.095 | | 1.047 | 1.145 |
|  | QTcFra | 0.001 | 6.832 | 2.177 | 21.443 | QTcB | 0.073 | 4.435 | | 0.869 | 22.622 |
|  | QTcH | 0.121 | 0.332 | 0.082 | 1.338 | QTcD | 0.542 | 0.582 | | 0.102 | 3.311 |
| Step 6 | Heart Rate | 0.076 | 1.022 | 0.998 | 1.047 | Heart Rate | 0.270 | 1.018 | | 0.986 | 1.051 |
|  | Age | 0.002 | 1.051 | 1.019 | 1.085 | Age | <0.001 | 1.095 | | 1.047 | 1.145 |
|  | **QTcFra** | **0.004** | **3.079** | **1.432** | **6.620** | **QTcB** | **0.032** | **2.917** | | **1.093** | **7.780** |

ULN=upper limit of normal, HR= Hazard Ratio, CI= confidence interval, QTc= Corrected QT interval, Bazett’s correction formula (QTcB), Fridericia’s correction formula (QTcFri), Dmitrienko’s correction formula (QTcD), Framingham’s correction formula (QTcFra), Hodges’s correction formula (QTcH), Rautaharju’s correction formula (QTcR).
